# Promoter region methylation does not account for the frequent loss of expression of the *Fas* gene in colorectal carcinoma

**DOI:** 10.1054/bjoc.1999.0889

**Published:** 1999-12-08

**Authors:** L M Butler, A Dobrovic, T Bianco, P A Cowled

**Affiliations:** Departments of Surger, and Medicine, The University of Adelaide, and Haematology-Oncology, The Queen Elizabeth Hospital, Woodville, South Australia, 5011, Australia

**Keywords:** CpG island, methylation, gene silencing, apoptosis

## Abstract

Expression of the apoptosis-promoting *Fas* gene is frequently reduced or lost during the development of colorectal carcinoma. However, loss of heterozygosity at the *Fas* locus or *Fas* gene rearrangements do not account for the loss of expression of *Fas*, raising the possibility that methylation of the *Fas* promoter may inhibit gene expression in colorectal carcinomas. We have examined the *Fas* promoter region CpG island for evidence of hypermethylation in colorectal tumours. Forty-seven specimens of colorectal adenoma and carcinoma, as well as six samples of normal colonic mucosa, were examined by Southern blotting for methylation at *Hpa* II and *Cfo* I sites in this region. No methylation was detected in any of the specimens, suggesting that hypermethylation is not primarily responsible for the loss of expression of the *Fas* gene during colorectal tumorigenesis. © 2000 Cancer Research Campaign


*British Journal of Cancer* (2000) **82**(1), 131–135
© 2000 Cancer Research Campaign
Article no. bjoc.1999.0889

### Promoter region methylation does not account for the frequent loss of expression of the *Fas* gene in colorectal carcinoma

**LM Butler[1], A Dobrovic[2,3], T Bianco[2,3] and PA Cowled[1]**

Departments of [1]Surger and [2]Medicine, The University of Adelaide, and [3]Haematology-Oncology, The Queen Elizabeth Hospital, Woodville, South Australia 5011, Australia

**Summary** Expression of the apoptosis-promoting *Fas* gene is frequently reduced or lost during the development of colorectal carcinoma. However, loss of heterozygosity at the *Fas* locus or *Fas* gene rearrangements do not account for the loss of expression of *Fas*, raising the possibility that methylation of the *Fas* promoter may inhibit gene expression in colorectal carcinomas. We have examined the *Fas* promoter region CpG island for evidence of hypermethylation in colorectal tumours. Forty-seven specimens of colorectal adenoma and carcinoma, as well as six samples of normal colonic mucosa, were examined by Southern blotting for methylation at *Hpa*II and *Cfo*I sites in this region. No methylation was detected in any of the specimens, suggesting that hypermethylation is not primarily responsible for the loss of expression of the *Fas* gene during colorectal tumorigenesis. © 2000 Cancer Research Campaign

**Keywords:** CpG island; methylation; gene silencing; apoptosis

There is increasing evidence that de novo methylation of promoter-associated CpG islands, contributes to the alteration of gene expression in cancer (reviewed by Baylin et al, 1998). During the development of colon cancer, methylation of CpG islands has been reported in a number of genes including the calcitonin gene (Silverman et al, 1989), the oestrogen receptor gene (Issa et al, 1994), the mismatch repair gene *hMLH*1 (Kane et al, 1997), the *MyoD* gene (*Myf*-3) (Iacopetta et al, 1997) and the *APC* gene (Hiltunen et al, 1997). More recently, a progressive increase in methylation was also detected at some of these loci in normal colonic mucosa as a consequence of aging (Ahuja et al, 1998). In the studies that examined gene expression, methylation of the CpG islands was associated with gene silencing.

In the colon, apoptosis contributes to the homeostasis of the epithelial layer of the mucosa, which has a rapid rate of cell turnover (Hall et al, 1994). Apoptosis is also responsible for the removal of colonocytes with potentially oncogenic DNA damage. Resistance of colonocytes to apoptosis may allow hyperproliferation, accumulation of oncogenic mutations and prevent killing of malignant cells by chemotherapeutic agents. Abnormal patterns of expression of a number of apoptosis-related genes have been reported in both benign and malignant colonic tumours (reviewed in Butler et al, 1999); however, molecular mediators of resistance to apoptosis remain to be identified.

The Fas antigen is a widely expressed cell surface receptor. Ligation of Fas by its endogenous ligand or by agonistic antibodies, triggers rapid apoptosis (Trauth et al, 1989; Yonehara et al, 1989). The epithelial layer of the normal colonic mucosa expresses Fas protein at high levels from the bottom of the crypts to the luminal surface (Leithauser et al, 1993; Moller et al, 1994). Expression of Fas in the colon is progressively reduced during the transformation of normal epithelium to benign neoplasms, adenocarcinomas and ultimately, to metastases (Leithauser et al, 1993; Moller et al, 1994). Loss of Fas activity could be a contributing factor to the reduction in apoptotic capacity of colonic carcinomas. In a previous study (Butler et al, 1998), we showed that the loss of expression of Fas protein was reflected in a loss of *Fas* mRNA in the majority of samples of colorectal carcinoma. However, allelic loss of the *Fas* gene was a relatively rare event, being detected in only 16% of carcinomas and reflecting the rate of loss of the entire chromosome arm, 10 q, in colorectal carcinoma (Vogelstein et al, 1989). Similarly, gross gene rearrangements were not detected in any of the colorectal carcinomas, raising the possibility that epigenetic events, including DNA methylation, could be responsible for the loss of expression of Fas.

The human *Fas* gene contains a 650 bp GC-rich CpG island spanning the 5′ regulatory region and the first exon (Behrmann et al, 1994; Cheng et al, 1995; Rudert et al, 1995), suggesting that transcription of the gene may be regulated by methylation of the CpG cytosine residues. The aim of this study was to determine the methylation status of the promoter and exon 1 of the *Fas* gene in DNA isolated from colorectal tumours with varying levels of expression of Fas mRNA.

*Received 22 February 1999*
*Revised 22 June 1999*
*Accepted 7 July 1999*

*Correspondence to:* PA Cowled

### MATERIALS AND METHODS

### Patients and samples

Forty-seven specimens of primary colonic or rectal tumours, consisting of six adenomas and 41 carcinomas plus six samples of macroscopically normal colonic mucosa, were obtained with informed consent from 44 patients undergoing colonic resections. The Dukes grades of the carcinomas are shown in Table 1.

**Table 1** Clinical stages of carcinomas analysed for methylation status of the *Fas* gene

| Dukes' grade | Number of specimens |
|---|---|
| A | 3 |
| B | 23 |
| C | 12 |
| D | 2 |
| Unclassified | 1 |

### Construction of a Fas Probe

PCR primers 5Prom1 (5′-TCCTGTACCCAGGCAGGAC) and 3Prom2 (5′-ATCCCCGGGACTAAGACGG) were designed to amplify a 655 base pair (bp) region spanning part of the promoter and exon one of the human *Fas* gene (Figure 1). The probe was amplified from normal genomic DNA by an initial denaturation of 5 min at 94°C, followed by 35 cycles of denaturation for 1 min at 94°C, annealing for 1 min at 58°C and extension for 90 s at 72°C; with a final extension of 5 min at 72°C. The PCR product was cloned into the pGEM-T plasmid vector (Promega, Madison, WI, USA) and the identity of the product confirmed by DNA sequencing. To generate a probe for Southern blotting, the *Fas* gene insert was amplified from the Fas-pGEM plasmid using 5Prom1 and 3Prom2 primers, under the same amplification conditions.

### Southern blotting using methylation-sensitive restriction enzymes

Ten micrograms of genomic DNA, isolated from colorectal tumours or normal mucosa, was digested with *Taq*I (New England Biolabs, Beverley, MA, USA), for 3 h or overnight at 65°C. DNA was ethanol-precipitated before re-digesting with *Hpa*II, *Msp*I (New England Biolabs) or *Cfo*I (Boehringer Mannheim). Digests were electrophoresed through 1.8% or 1.5% agarose gels and blotted onto Genescreen Plus membranes (Dupont, Boston, MA, USA). Membranes were hybridized with the $^{32}$P-labelled probe for 16 h at 42°C in 50% formamide, washed to a final stringency of 0.1 × SSC (standard saline citrate) and 0.1% sodium dodecyl sulphate (SDS) at 68°C and the hybridization signals detected by autoradiography.

### RESULTS

The methylation status of the 5′ regulatory region and exon 1 of the *Fas* gene was initially examined by Southern blotting of DNA following digestion with the restriction enzymes *Msp*I and *Hpa*II. Both of these enzymes recognize the same DNA sequence (CCGG); however, *Hpa*II will not cut the sequence if the internal cytosine residue is methylated. Six *Hpa*II/*Msp*I sites are present in the region spanning the three putative transcriptional start sites within the first exon of the *Fas* gene (Cheng et al, 1995) (Figure 1). A second enzyme, *Cfo*I, was also used to assess the methylation status of a further five CpG sites in this region (Figure 1). *Cfo*I cleaves the sequence GCGC, but is inactive if the central C is methylated. A *Fas* gene probe was generated by polymerase chain reaction (PCR) amplification and its identity confirmed by sequencing (Cheng et al, 1995).

The DNA was initially digested with *Taq*I, which flanked five of the *Hpa*II sites as well as four of the *Cfo*I sites. The recognition sequence of *Taq*I (TCGA) contains a CpG dinucleotide; however, *Taq*I is not methylation-sensitive (Streeck, 1980). Digestion of normal DNA (isolated from peripheral blood lymphocytes) with the flanking enzyme, *Taq*I, alone, produced a band of 1 kb when hybridized with the Fas probe (Figures 2 and 3). A second band of approximately 5 kb in size was also detected, arising from binding of the probe to sequences upstream of the *Taq*I site in the *Fas* promoter.

When the *Taq*I-digested normal DNA was digested with *Msp*I, bands of 300 bp and 115 bp were observed, as well as the 5 kb

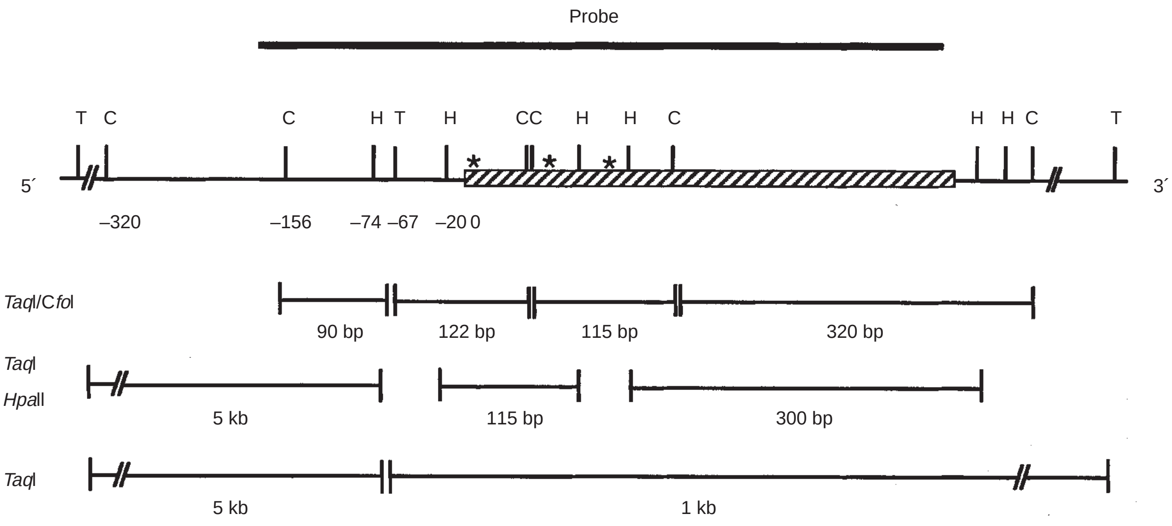

**Figure 1** Restriction map of the 5′ regulatory region and exon 1 of the human *Fas* gene. T = *Taq*I site, H = *Hpa*II site, C = *Cfo*I site. The asterisks denote the major three transcription start sites (Cheng et al, 1995) and the hatched box denotes exon 1. The sizes of the major DNA restriction fragments are shown underneath the map and the location of the 655 bp *Fas* probe is shown above the map. The 5′ end of the probe detects an approximately 5 kb *Taq*I fragment. Only the 1957 bp section directly upstream of the first transcription start site has been sequenced (Cheng et al, 1995, Rudert et al, 1995)

© 2000 Cancer Research Campaign

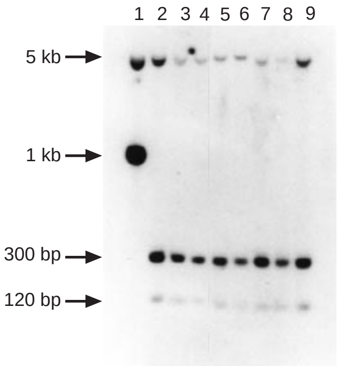

**Figure 2** Southern analysis of the *Fas* gene promoter from colorectal tumours for changes in methylation. The blot was hybridized with a 655 bp probe spanning five *Hpa*II sites, in the promoter and first intron of the human *Fas* gene. Lane 1 contains normal genomic DNA digested with *Taq*I, lane 2 contains the same DNA digested with *Taq*I and *Msp*I, while lanes 3–9 contain individual samples of DNA from colonic tumours, digested with *Taq*I and *Hpa*II

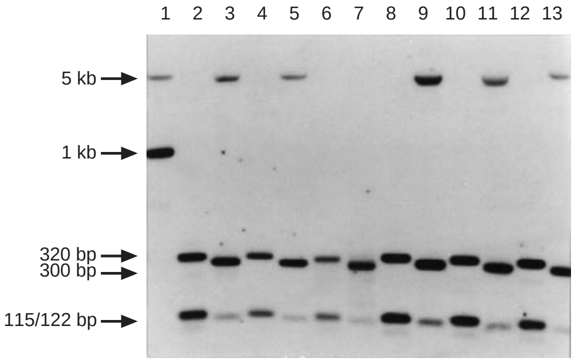

**Figure 3** Southern analysis of the *Fas* gene promoter from colorectal tumours for changes in methylation. The blot was hybridized with a 655 bp probe spanning five *Hpa*II sites and four *Cfo*I sites in the promoter and first intron of the human *Fas* gene. Lane 1 contains genomic DNA digested with *Taq*I only and lanes 2–13 contain samples of DNA from colonic tumours, digested with *Taq*I and *Cfo*I (odd numbered lanes) or *Taq*I and *Hpa*II (even numbered lanes)

band previously observed following digestion with *Taq*I alone (Figure 2). According to a restriction map of the Fas promoter and exon 1, digestion of the 1 kb *Taq*I fragment with *Msp*I and probing with the 655 bp Fas probe, should produce DNA fragments of 300, 115, 47, 43 and 28 bp (Figure 1). The smallest three fragments could not be detected by Southern analysis. The methylation status of the 1 kb region could then be determined by digestion of DNA with *Taq*I and *Hpa*II. If the sequence was fully methylated, *Hpa*II would not cut the DNA and the banding pattern would be the same as that of *Taq*I alone. An unmethylated sequence would be completely digested by *Hpa*II and the banding pattern would be the same as digestion with *Taq*I and *Msp*I. A partially methylated sequence would give rise to bands of intermediate size.

There are no *Msp*I sites in the 1957 bp of known sequence upstream of the first transcription start site (Cheng et al, 1995; Rudert et al, 1995). Since there was no detectable change in the size of the 5 kb band following digestion with both *Taq*I and *Msp*I, it can be deduced that there are probably no *Msp*I sites in the undefined upstream sequence (Figure 1).

Forty-eight samples of DNA isolated from colonic carcinomas plus six adenomas were analysed for methylation of the *Hpa*II sites. These samples included a range of tumour grades (Table 1). Two of the patients had both an adenoma and carcinoma analysed and one patient had both a primary tumour and a metastatic deposit. Digestion of the DNA samples with *Taq*I and *Hpa*II produced the same banding pattern as digestion with *Taq*I and *Msp*I. The *Hpa*II sites were therefore not methylated in any of the tumour samples analysed (Figure 2). There was also no evidence of Fas methylation in six samples of DNA isolated from normal colorectal mucosa.

Nineteen of the carcinomas were also assessed for methylation at the *Cfo*I sites (Figure 3), however, none of these sites were methylated in any of the tumours tested. Digestion of DNA with *Taq*I and *Cfo*I gives rise to bands of 320, 122, 115 and 90 bp in size. The 90-bp band was detected in the more-heavily-loaded tracks and the 122 and 115 bp bands coincided on the blots. No 5 kb band was detected in *Cfo*I/*Taq*I-digested DNA, confirming that the *Cfo*I site located 156 bp upstream of the first transcription start site is unmethylated in all samples tested. There is a further *Cfo*I site 320 bp upstream of the first transcription start site (Figure 1) but the methylation status of this site can not be analysed as the probe does not cover this region.

Seventeen of the tumours used in this study have previously been analysed by Northern blotting for expression of Fas mRNA (Butler et al, 1998). Nine of the tumours expressed normal levels of Fas mRNA, five had reduced levels and, in two samples, Fas mRNA could not be detected. This study probably underestimated the loss of *Fas* as most tumours also contained normal tissue.

### DISCUSSION

Cancer arises from the accumulation of multiple genetic and epigenetic events in cellular DNA. These alterations can cause the aberrant expression of genes involved in the regulation of cell death, adhesion and proliferation. Methylation of CpG residues in the 5′ regulatory regions of tumour suppressor genes may be a mechanism by which cellular proliferation can be deregulated without genetic mutations (reviewed in Baylin et al, 1998). In colon cancer, alterations in global methylation patterns are among the earliest abnormalities to occur during the development of the disease (Goelz et al, 1985). The studies presented above indicate that the *Fas* gene promoter is consistently unmethylated in colorectal tumours and normal mucosa. This suggests that methylation of the Fas promoter is not involved in the transcriptional silencing of the *Fas* gene in colon tumours.

Screening samples of DNA for cytosine methylation using restriction enzymes only examines a proportion of the total CpG dinucleotides in a CpG island. There are 31 CpG sites in the 655 bp region of the *Fas* promoter and exon 1 spanned by the *Fas* probe in the present study (Cheng et al, 1995). The *Hpa*II and *Cfo*I sites examined in the present study represent nine of them, or 29%. One of the *Hpa*II sites in the promoter region is 20 bp upstream of the first transcription start site. Any CpG methylation at this site might interfere with binding of RNA polymerase and inhibit transcription of the Fas gene. It is possible that de novo methylation of the *Fas* promoter region does not involve all CpG sites and that selective methylation of the CpG island is sufficient for the regulation of transcription factor binding. Further studies using other

*© 2000 Cancer Research Campaign* 

methodologies, including genomic sequencing using bisulphite modification, which determines the methylation status of all cytosines in a sequence (Frommer et al, 1992), are required to totally exclude methylation that is restricted to a small region.

Other mechanisms must therefore be considered to account for the frequent loss of expression of the Fas protein in colorectal carcinoma. One mechanism for loss of Fas expression is that expression or function of an essential transcription factor has been altered. The promoter region of the *Fas* gene contains binding sites for several transcriptional regulatory factors, including c-myb, p53, NF-κB and Sp-1 (Behrmann et al, 1994; Cheng et al, 1995; Rudert et al, 1995). An essential role has recently been demonstrated for a composite Sp1/NF-κB-binding site in the Fas promoter, in activating the expression of Fas mRNA in Jurkat cells (Chan et al, 1999). Futher studies will be required to investigate the hypothesis that defects in Sp1/NF-κB activity could account for loss of expression of Fas in colorectal carcinomas.

A potential candidate for the regulation of the activity of the *Fas* gene is p53, which, when expressed in tumour cells, induces the expression of Fas mRNA (Owen-Schaub et al, 1995). Expression of wild-type p53 in colon cancer cells renders them sensitive to Fas-mediated apoptosis (Tamura et al, 1995) and certain genotoxic treatments induce cell surface expression of Fas only in cells with wild-type *p53* genes (Matsumoto et al, 1996; Muller et al, 1997; Reap et al, 1997; Reinke and Lozano, 1997; Sheard et al, 1997). Taken together, these studies indicate that the *Fas* gene is a target of p53-mediated transactivation. Inactivation of the *p53* gene by mutation and/or allelic loss occurs in up to 75% of colorectal carcinomas (Baker et al, 1990), a similar frequency to loss of Fas expression (Moller et al, 1994; Butler et al, 1998). Loss of Fas expression may therefore be a consequence of p53 inactivation in most colorectal tumours. The activity of histone acetyltransferases and deacetylases is also vital in regulating gene regulation (reviewed in Kuo and Allis, 1998) and alterations in the activity of these enzymes might also play a role in the silencing of the *Fas* gene in colorectal carcinoma. Further studies are required to define the molecular mechanisms controlling expression of the *Fas* gene and to determine the nature and significance of the defects in Fas expression in colorectal carcinoma.

### ACKNOWLEDGEMENTS

LMB was supported by an Australian Postgraduate Research Scholarship and by supplementary funding from The Queen Elizabeth Hospital Research Foundation. TB was supported by an Australian Postgraduate Research Scholarship. We wish to thank Mr Peter Hewett for providing the clinical samples.

### REFERENCES

Ahuja N, Li Q, Mohan AL, Baylin SB and Issa J-PJ (1998) Aging and DNA methylation in colorectal mucosa and cancer. *Cancer Res* **58**: 5489–5494

Baker SJ, Markowitz S, Fearon ER, Willson JKV and Vogelstein B (1990) Suppression of human colorectal carcinoma cell growth by wild-type p53. *Science* **249**: 912–915

Baylin SB, Herman JG, Graff JR, Vertino PM and Issa J-P (1998) Alterations in DNA methylation: a fundamental aspect of neoplasia. *Adv Cancer Res* **72**: 141–196

Behrmann I, Walczak H and Krammer PH (1994) Structure of the human APO-1 gene. *Eur J Immunol* **24**: 3057–3062

Butler LM, Hewett PJ, Butler WJ and Cowled PA (1998) Down-regulation of Fas gene expression in colon cancer is not a result of allelic loss or gene rearrangement. *Br J Cancer* **77**: 1454–1459

Butler LM, Hewett PJ, Fitridge RA and Cowled PA (1999) Deregulation of apoptosis in colorectal cancer: theoretical and therapeutic implications. *Aust NZ J Surg* **69**: 88–94

Chan H, Bartos DP and Owen-Schaub LB (1999) Activation-dependent transcriptional regulation of the human Fas promoter requires NF-kappaB p50-p65 recruitment. *Mol Cell Biol* **19**: 2098–2108

Cheng J, Liu C, Koopman WJ and Mountz JD (1995) Characterization of human *Fas* gene. *J Immunol* **154**: 1239–1245

Frommer M, McDonald LE, Millar DS, Collis CM, Watt F, Grigg GW, Molloy PL and Paul CL (1992) A genomic sequencing protocol that yields a positive display of 5-methylcytosine residues in individual DNA strands. *Proc Natl Acad Sci USA* **89**: 1827–1831

Goelz SE, Vogelstein B, Hamilton SR and Feinberg AP (1985) Hypomethylation of DNA from benign and malignant human colon neoplasms. *Science* **228**: 187–190

Hall PA, Coates PJ, Ansari B and Hopwood D (1994) Regulation of cell number in the mammalian gastrointestinal tract: the importance of apoptosis. *J Cell Sci* **107**: 3569–3577

Hiltunen MO, Alhonen L, Koistinaho J, Myöhänen S, Pääkkönen M, Marin S, Kosma V-M and Jänne J (1997) Hypermethylation of the *APC* (adenomatous polyposis coli) gene promoter region in human colorectal carcinoma. *Int J Cancer* **70**: 644–648

Iacopetta BJ, Harmon D, Spagnolo DV, House AK and Kay PH (1997) Hypermethylation of the *Myf*-3 gene in human colorectal cancer. *Anticancer Res* **17**: 429–432

Issa J-P, Ottaviano YL, Celano P, Hamilton SR, Davidson NE and Baylin SB (1994) Methylation of the oestrogen receptor CpG island links ageing and neoplasia in human colon. *Nat Genet* **7**: 536–540

Kane MF, Loda M, Gaida GM, Lipman J, Mishra R, Goldman H, Jessup JM and Kolodner R (1997) Methylation of the *hMLH1* promoter correlates with lack of expression of hMLH1 in sporadic colon tumors and mismatch repair-defective human tumor cell lines. *Cancer Res* **57**: 808–811

Kuo MH and Allis CD (1998) Roles of histone acetyltransferases and deacetylases in gene regulation. *Bioessays* **20**: 615–626

Leithäuser F, Dhein J, Mechtersheimer G, Koretz K, Brüderlein S, Henne C, Schmidt A, Debatin K-M, Krammer PH and Möller P (1993) Constitutive and induced expression of APO-1, a new member of the nerve growth factor/tumor necrosis factor receptor superfamily, in normal and neoplastic cells. *Lab Invest* **69**: 415–429

Matsumoto Y, Hayakawa A, Tamada Y, Mori H and Ohashi M (1996) Upregulated expression of Fas antigen on cultured human keratinocytes with induction of apoptosis by cisplatin. *Arch Dermatol Res* **288**: 267–269

Möller P, Koretz K, Leithauser F, Brüderlein S, Henne C, Quentmeier A and Krammer PH (1994) Expression of APO-1 (CD95), a member of the NGF/TNF receptor superfamily, in normal and neoplastic colon epithelium. *Int J Cancer* **57**: 371–377

Muller M, Strand S, Hug H, Heinemann E-M, Walczak H, Hofmann WJ, Stremmel W, Krammer PH and Galle PR (1997) Drug-induced apoptosis in hepatoma cells is mediated by the CD95 (APO-1/Fas) receptor/ligand system and involves activation of wild-type p53. *J Clin Invest* **99**: 403–413

Owen-Schaub LB, Zhang W, Cusack JC, Angelo LS, Santee SM, Fujiwara T, Roth JA, Deisseroth AB, Zhang W-W, Kruzel E and Radinsky R (1995) Wild-type human p53 and a temperature-sensitive mutant induce Fas/APO-1 expression. *Mol Cell Biol* **15**: 3032–3040

Reap EA, Roof K, Maynor K, Borrero M, Booker J and Cohen PL (1997) Radiation and stress-induced apoptosis: a role for Fas/Fas ligand interactions. *Proc Natl Acad Sci USA* **94**: 5750–5755

Reinke V and Lozano G (1997) The p53 targets *mdm2* and *Fas* are not required as mediators of apoptosis in vivo. *Oncogene* **15**: 1527–1534

Rudert F, Visser E, Forbes L, Lindridge E, Wang Y and Watson J (1995) Identification of a silencer, enhancer, and basal promoter region in the human CD95 (Fas/APO-1) gene. *DNA Cell Biol* **14**: 931–937

Sheard MA, Vojtesek B, Janakova L, Kovarik J and Zaloudik J (1997) Up-regulation of Fas (CD95) in human $p53^{wild\text{-}type}$ cancer cells treated with ionizing radiation. *Int J Cancer* **73**: 757–762

Silverman AL, Park J-G, Hamilton SR, Gazdar AF, Luk GD and Baylin SB (1989) Abnormal methylation of the calcitonin gene in human colonic neoplasms. *Cancer Res* **49**: 3468–3473

Streeck RE (1980) Single-strand and double-strand cleavage at half-modified and fully modified recognition sites for the restriction nucleases *Sau3A* and *Taq*I. *Gene* **12**: 267–275

Tamura T, Aoyama N, Saya H, Haga H, Futami S, Miyamoto M, Koh T, Ariyasu T, Tachi M, Kasuga M and Takahashi R (1995) Induction of Fas-mediated apoptosis in p53-transfected human colon carcinoma cells. *Oncogene* **11**: 1939–1946

 © 2000 Cancer Research Campaign

Trauth BC, Klas C, Peters AMJ, Matzku S, Möller P, Falk W, Debatin K-M and Krammer PH (1989) Monoclonal antibody-mediated tumor regression by induction of apoptosis. *Science* **245**: 301–304

Vogelstein B, Fearon ER, Kern SE, Hamilton SR, Preisinger AC, Nakamura Y and White R (1989) Allelotype of colorectal carcinomas. *Science* **244**: 207–211

Yonehara S, Ishii A and Yonehara M (1989) A cell-killing monoclonal antibody (anti-Fas) to a cell surface antigen co-down-regulated with the receptor of tumor necrosis factor. *J Exp Med* **169**: 1747–1756

© 2000 Cancer Research Campaign
